# Be an ethicist not a stranger! The critical patient stuck between the right to evidence-based medicine, informed consent, and social duty. Are we still “strangers at the bedside”?

**DOI:** 10.1186/s44158-023-00110-w

**Published:** 2023-08-04

**Authors:** Agnese Accogli, Marco Vergano

**Affiliations:** 1 Department of Surgical Science, University of Turin, Turin, Italy; 2 Department of Anesthesia and Intensive Care, San Giovanni Bosco Hospital, Turin, Italy

**Keywords:** Research ethics, Informed consent, Evidence-based medicine, Ethics education

To introduce the current complex scenario of emergency research ethics, let us borrow some crucial thoughts from a L. Eisenberg article dating back to 1977. In his piece, the author highlighted perfectly how while “the critics of research are often exquisitely aware of the dangers in an experiment,” they really seem to be “surprisingly naive about the extent to which medical practice rests on custom rather than evidence,” failing to recognize the value of trials in determining “whether what is traditional does harm rather than good.” Eisenberg also pointed out that “the major barriers to the treatment of life-threatening disease stem not from failing to use what we know but from not knowing what to use” [1].

Nowadays, these words sound more relevant than ever. Medical progress has reached the status of moral obligation owed to society. Many authors recognize the social value of research in its contribution to the so called *public good*. For someone, there is a real diktat both in conducting and in participating in medical research. The *ethical catastrophe*, outlined by many as a direct result of hampered research, leads to the strict warning not to hinder the biomedical progress, achievable through experimentation [1, 2].

However, research on human beings is still paying the debt left behind by the barbarities that blemished its past, with some clinical settings resulting more damaged than others. A certain victim is critical care medicine. In order to protect the vulnerable patients, the critical ones, not able to express a consent, have been excluded from trials for a very long time. As a consequence, lots of current standards of care are not evidence based [2, 3].

Scientific community repeatedly asked for a change in international regulations, to updates them and (to) uniform the modus operandi. To date, the prerequisites necessary to conduct a trial in the emergency field are very restrictive and lead to discrepancies between the same vulnerable patients. Just to list few examples, let us remind you about the *minimal risk grey zone* and the misleading concept of *direct benefit* [4–7].

Without a doubt, the EU General Data Protection Regulation, the Clinical Trials Regulation, and the latest CIOMS Ethical Guidelines made some progresses, but some topics remain debated and unclear. International multicenter clinical trials still have to cope with the differences allowed by national legislation, leading to a “fragmented landscape” of research [8, 9].

Wondering about the role of the intensivist in this scenario, we must go back in time. In 1966 Henry K. Beecher, professor of research and teaching in anesthesia at Harvard Medical School, wrote his famous article, *Ethics and Clinical Research*, published in the *NEJM*, condemning a series of unethical trials conducted in the USA. He stated that for an ethical research two elements are essential. First, informed consent. Then, the “safeguard provided by the presence of an intelligent, informed, conscientious, compassionate, responsible investigator.”

Concerning informed consent, the horizon is getting every day wider. The international cooperation leading to shared data and bio-specimens biobanking is generating new dynamic types of consent that lead to a bunch of new questions dealing with incompetent patients [10, 11].

Moreover, the adjective “informed” usually results in extremely technical and long forms, grown up in a defensive medicine environment rather than under ethical pressure. Even the Italian National Bioethics Committee has underlined that informed consent itself is neither a proof of ethicity nor of scientificity and that ethics committees have the crucial role of judging protocols [12].

So, beyond the cornerstone of informed consent, aware of all its limits, stands the ethics committee, with its role of supervisor of ongoing trials and its educational tasks. But, at least in Italy, the already overwhelmed committees are now facing a relevant downsizing after the recent Italian law provisions on the reform of the REC network, in order to comply with the Regulation (EU) No 536/2014, with a transition “from an ethics review system with strong local roots to a centralized one” [2].

Still standing at the end of the road, the aware and critical thinking researcher that Beecher wished gains value. But recent national surveys show that in 2017 the 24% of study coordinators were not informed about the 2014 European research regulations yet, and the 92% stated that there was no continuing education in this field in their centers of research [13]. Data from 2019 stressed the lack of interaction between medical staff and local ethics committees on both clinical and research topics [14], proving that a great work is still needed.

In his book *Strangers at the bedside*, referring to World War II time, David J. Rothman wrote: “researchers and subjects were more likely to be strangers to each other, with no necessary sense of shared purpose or objective.” Today, this is not acceptable anymore. Intensivists must aim at becoming that model of safeguard already described, building a new concept of research participation based on solidarity, and we cannot overlook tailored ethics education to achieve this goal.

Let us conclude with a breath of fresh air. In front of all that lacks in terms of knowledge and awareness, we must take a look into the past. Digging back into Beecher’s history, you will find something really controversial. The reason why he has been defined “a late-blooming ethicist” [15]. Let us take this as a wish for all intensivists involved in research; a turning point is possible: be an ethicist not a stranger!

## Data Availability

Data sharing not applicable to this article as no datasets were generated or analyzed during the current study.
